# Scene construction in developmental amnesia: An fMRI study^☆^

**DOI:** 10.1016/j.neuropsychologia.2013.11.001

**Published:** 2014-01

**Authors:** Sinéad L. Mullally, Faraneh Vargha-Khadem, Eleanor A. Maguire

**Affiliations:** a Wellcome Trust Centre for Neuroimaging, Institute of Neurology, University College London, 12 Queen Square, London, WC1N 3BG, UK; b Developmental Cognitive Neuroscience Unit, Institute of Child Health, University College London, 30 Guilford Street, London, WC1N 1EH, UK

**Keywords:** Scene construction, Episodic memory, Future-thinking, Hippocampus, Semantic memory, Amnesia

## Abstract

Amnesic patients with bilateral hippocampal damage sustained in adulthood are generally unable to construct scenes in their imagination. By contrast, patients with developmental amnesia (DA), where hippocampal damage was acquired early in life, have preserved performance on this task, although the reason for this sparing is unclear. One possibility is that residual function in remnant hippocampal tissue is sufficient to support basic scene construction in DA. Such a situation was found in the one amnesic patient with adult-acquired hippocampal damage (P01) who could also construct scenes. Alternatively, DA patients’ scene construction might not depend on the hippocampus, perhaps being instead reliant on non-hippocampal regions and mediated by semantic knowledge. To adjudicate between these two possibilities, we examined scene construction during functional MRI (fMRI) in Jon, a well-characterised patient with DA who has previously been shown to have preserved scene construction. We found that when Jon constructed scenes he activated many of the regions known to be associated with imagining scenes in control participants including ventromedial prefrontal cortex, posterior cingulate, retrosplenial and posterior parietal cortices. Critically, however, activity was not increased in Jon's remnant hippocampal tissue. Direct comparisons with a group of control participants and patient P01, confirmed that they activated their right hippocampus more than Jon. Our results show that a type of non-hippocampal dependent scene construction is possible and occurs in DA, perhaps mediated by semantic memory, which does not appear to involve the vivid visualisation of imagined scenes.

## Highlights

• Scene construction is associated with intact hippocampal function.
• Patients with developmental amnesia (DA) and hippocampal lesions can construct scenes.
• DA patient Jon did not activate his hippocampi during fMRI while constructing scenes.
• Patients with DA may not truly visualise scenes but use semantic knowledge instead.

## 1 Introduction

Patients with bilateral hippocampal damage and concomitant amnesia are generally unable to construct and visualise spatially-coherent scenes in their mind's eye (Andelman et al., 2010, Hassabis et al., 2007, Klein et al., 2002, Mullally et al., 2012, Tulving, 1985; but see Squire et al., 2010, Maguire et al., 2010, for a response). It has been suggested that scene construction is required for episodic memory, imagining the future and spatial navigation, and that losing the ability to construct and visualise scenes may account for some of the symptomology of hippocampal amnesia (Hassabis and Maguire, 2007, Hassabis and Maguire, 2009, Maguire and Hassabis, 2011). However, there are patients with bilateral hippocampal damage and profound amnesia who seem able to construct scenes, which appears to challenge this view.

**Fig. 1:**
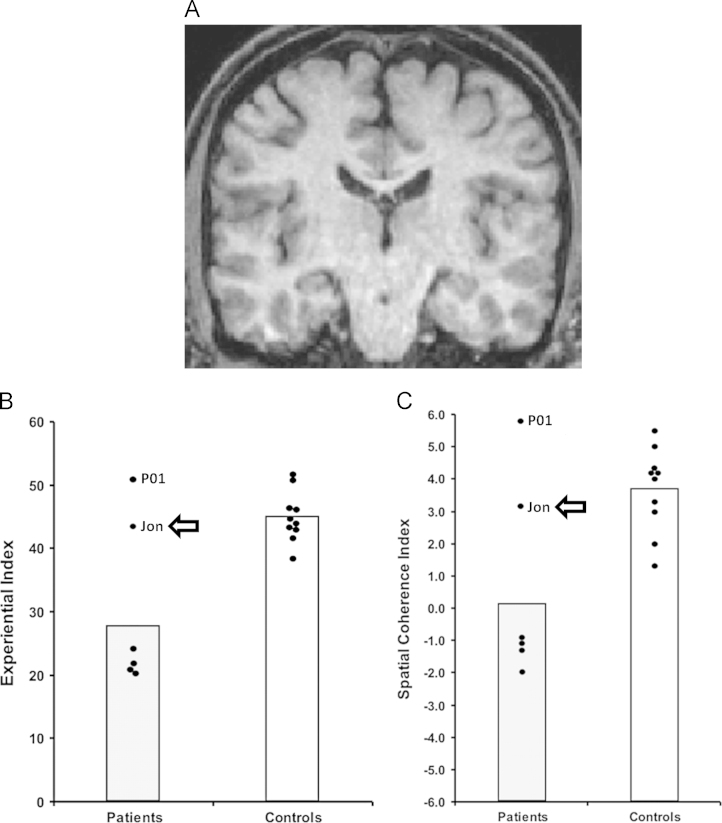
Patient Jon. (A) Shows a coronal view from Jon's MRI brain scan. (B) Scores on the scene construction Experiential Index (a measure of the overall richness of imagined scenes). (C) Scores on the scene construction Spatial Coherence Index (a measure of the spatial contiguousness of imagined scenes). Data are from the Hassabis et al. (2007) scene construction task, where each dot represents the data point of a patient with amnesia whose bilateral hippocampal damage was sustained in adulthood (*n*=5) and ten matched control participants. These include Patient P01, who is highlighted. The data points for Jon are highlighted with an arrow. Vertical bars signify means for each group. Jon performed similarly to the mean of the control participants and significantly better than the other patients with hippocampal damage and amnesia.

For instance, Hassabis et al. (2007) noted that one of their five amnesic patients (P01, called KN in Aggleton 2005, McKenna and Gerhand, 2002) could construct spatially-coherent scenes and describe personal future events, in marked contrast to the other four patients (Fig. 1). Using functional MRI (fMRI) it has recently been shown that P01 activated the remnant of his right hippocampus while constructing scenes (Mullally, Hassabis, & Maguire, 2012). Thus, P01 appeared to have sufficiently preserved function in his right hippocampal tissue to support basic scene construction, a finding that reinforces the role played by the hippocampus in scene construction.

The patients alluded to above acquired their hippocampal damage in adulthood. By contrast, developmental amnesia (DA) occurs following a hypoxic/ischemic incident perinatally or in early childhood, resulting in bilateral hippocampal pathology (Gadian et al., 2000, Isaacs et al., 2003, Vann et al., 2009, Vargha-Khadem et al., 2003). A distinguishing feature of DA compared to adult-onset amnesia is that the entire content of semantic memory and world knowledge, which is rich and age appropriate, has been accumulated *after* the onset of bilateral hippocampal pathology (Vargha-Khadem et al., 1997). Another distinguishing feature has recently been observed, namely, that DA patients appear able to construct scenes. This was first reported in patient Jon who, now an adult, is one of the best characterised DA patients (Vargha-Khadem et al., 1997). Jon sustained his hippocampal damage perinatally, with a volume loss of approximately 50% bilaterally (Gadian et al., 2000). He subsequently presented with symptoms of DA at an early age, and while severely amnesic for his personal life events, Jon has been able to acquire an impressive body of semantic knowledge (Vargha-Khadem et al., 1997). He could also successfully construct fictitious scenes and imagine future events (Maguire, Vargha-Khadem, & Hassabis, 2010; Fig. 1). Similarly, in a study involving 21 school-age patients with moderate memory impairment or DA from neonatal exposure to hypoxia-ischaemia, Cooper, Vargha-Khadem, Gadian, and Maguire (2011) observed intact scene construction relative to age-matched controls, as did Hurley, Maguire, and Vargha-Khadem (2011) (but see Kwan, Carson, Addis, & Rosenbaum, 2010) when testing another DA patient HC (DA E6 in Vargha-Khadem et al., 2003, Isaacs et al., 2003; DA 6 in Adlam, Malloy, Mishkin, & Vargha-Khadem, 2009), thus confirming that intact scene construction appears to be a consistent feature of DA.

The reason for the apparently preserved scene construction in DA is not clear, but understanding its neural substrate is critical for evaluating the role of the hippocampus in constructing scenes. In order to examine this issue further, we investigated patient Jon's scene construction ability using fMRI. One hypothesis is that, like P01, Jon (and other patients with DA), may be able to construct scenes because of residual function in remnant hippocampal tissue. If one considers the cases of Jon and P01, this seems plausible as both Jon and P01 share a number of similarities. Both are densely amnesic suffering from a profound impairment of episodic memory. Both have significant hippocampal volume loss (of approximately 50% bilaterally), but have nevertheless acquired semantic knowledge post-lesion (Jon: Vargha-Khadem et al., 1997; P01: McKenna & Gerhand, 2002), and activity in residual hippocampal tissue has been documented in both patients. In the case of Jon this was observed during an autobiographical memory recall task (Maguire, Vargha-Khadem, & Mishkin, 2001), while for P01, right hippocampal activity was observed during semantic learning (see Hassabis et al., 2007—Supplementary material) and scene construction (Mullally et al., 2012). It is therefore possible that Jon's remnant hippocampal tissue might be involved in supporting his seemingly preserved scene construction ability.

However, against this backdrop of commonalities, Jon differs from P01, and from healthy participants in an important way. While P01 (Hassabis et al., 2007, Mullally et al., 2012) and controls (Hassabis, Kumaran, & Maguire, 2007) described scene construction as effortless and natural, Jon reported that he found the process effortful, and describes it as something which he has had to train himself to accomplish (Maguire et al., 2010). It is therefore possible that while P01 and controls engage in similar scene construction processes, Jon's scene construction may be phenomenologically distinct and could be supported by a distinct neural network. Perhaps in DA, preserved semantic and world knowledge is sufficient to sustain a process akin to scene construction but which does not permit the vivid visualisation of spatially-coherent scenes, and which does not therefore depend on the hippocampus.

Based on behavioural data alone it is impossible to ascertain how similar to normal scene construction the DA patients' experiences are, given that they may never have truly imagined a scene, making the accuracy of their introspections difficult to interpret. However, examining the brain regions supporting their scene construction processes could offer some insights into this issue. Jon may activate the ‘core’ scene network (which includes the medial temporal lobes (MTL), ventromedial prefrontal cortex (VMPFC), posterior parietal cortex, precuneus, and retrosplenial cortex) similar to that activated by controls (Hassabis et al., 2007, Spreng et al., 2009, Summerfield et al., 2010) and P01 (Mullally et al., 2012). On the other hand, he may fail to display hippocampal or any MTL activation when he constructs scenes. This could be accompanied by normal activation of the rest of the core network, up-regulation of brain areas within the core network, or recruitment of additional brain areas that are not usually engaged by control participants during scene construction.

Using an fMRI paradigm adapted from Hassabis et al. (2007) and identical to that used in the study of P01 (Mullally et al., 2012) we examined the brain areas supporting Jon's scene construction. At the time of testing, Jon was similar in age to the healthy controls scanned previously by Hassabis et al. (2007), enabling us to draw comparisons between them. In addition, and despite their differing aetiologies and ages, Jon and P01 had so much in common that it was also of interest to make comparisons between the two patients. We hypothesised that, unlike the controls and P01, the non-hippocampal components of the ‘core’ network would support Jon's scene construction, because we suspected his effortful construction did not rely on true hippocampal-dependent visualisation of spatially-coherent scenes.

## 2 Materials and methods

### 2.1 Participants

#### 2.1.1 Patient Jon

Patient Jon, who was 32 years old at time of testing, is a well-documented case of DA. Briefly, he was born prematurely at 26 weeks of gestation. He weighed less than 1 kg, suffered breathing problems and during his first 6 weeks of life required intubation and positive pressure ventilation for severe apnoeic attacks (Gadian et al., 2000). He subsequently showed steady improvement and normal development, but by the age of five memory problems were noted, and have since continued to be prominent. Direct measurement of Jon's MRI scans in adulthood indicated a reduction of ~50% in the volume of left and right hippocampi, with no evident pathology in the rest of the MTL (Gadian et al., 2000, Vann et al., 2009). Consistent with his hippocampal abnormality, Jon has difficulty in reliably finding his way. He also tends to forget where belongings are normally kept, has problems remembering everyday events such as TV programmes just seen and is typically unable to give a detailed account of his activities earlier in the day.

#### 2.1.2 Control participants

Our aim was to examine Jon as a single case to ascertain if his preserved ability to construct fictitious scenes was accompanied by engagement of his remnant hippocampal tissue during fMRI. However, we also performed comparisons between Jon and a group of control participants in order to examine the wider set of brain areas engaged during scene construction. There were 21 control participants (10 males; mean age 24.8 years (SD 3.8); age range 18–31 years) whose results were reported previously by Hassabis et al. (2007).

#### 2.1.3 Patient P01

We were also interested in comparing Jon with patient P01. His case has been described in detail elsewhere (Aggleton 2005, McKenna and Gerhand, 2002, Mullally et al., 2012). To summarise, this male, right-handed former industrial biochemist, who was 51 years old at the time of his fMRI scan, had contracted meningeo-encephalitis at the age of 34 and then recurrent meningitis. He was left without useful motor function below T12, loss of vision in the lower visual field, and severe amnesia. While his structural MRI scans showed bilateral abnormalities in the occipital lobes, the main locus of volume reduction was in the hippocampi (reduced by 48.8% on the left and 46.2% on the right).

All participants performed the same tasks (with minor modifications—see details below), in the same MRI scanner, using the same image acquisition parameters, and identical data analysis protocol. All gave informed written consent to participation in accordance with the local research ethics committee.

### 2.2 Tasks and procedure

The control participants (reported in Hassabis et al., 2007) performed six trial types: construct a novel fictitious scene, construct a novel acontextual object, recall a recent autobiographical memory, recall a previously viewed acontextual object, recall a previously imagined fictitious scene, recall a previously imagined acontextual object. Each trial had an identical structure. First, participants were presented with a trial cue (e.g. ‘IMAGINE…Standing on a crowded platform of a train station’). The trial cue remained on screen for 5.5 s and was then replaced by a “Close your eyes and imagine” instruction. Participants then closed their eyes and began to visualise as vividly as possible. During this 16 s visualisation period participants were required to focus on the memory, scene or object they were recalling or imagining. A 1 s audio tone signalled the end of the visualisation period (at which point participants had to open their eyes) and the start of the ratings phase. Using an MR-compatible five-button keypad, participants scored their just-visualised memory, scene or object across four ratings: difficulty (how difficult was the trial: 1, very easy…5, very hard), vividness (salience of the imagery: 1, not vivid…5, very vivid), spatial coherence (contiguousness of the spatial context: 1, an isolated object…5, a contiguous scene), and memory (how much like a memory the visualised scene or object was: 1, nothing at all like a memory…5, exactly like a memory). They had 4.5 s to respond for each rating. This was followed by a 1 s period of rest before the cue for the next trial was presented. A baseline control condition was also included. Here participants had to imagine a white cross on a black background. This was followed by one rating (‘how focused on the cross did you manage to stay: 1, not at all focused…5, very focused). Detailed instructions and multiple practice trials were given to participants prior to scanning to ensure that they were confidently able to adhere to task requirements. Training included how to imagine the single, novel objects in the mind's eye (in response to an on-screen cue, e.g. “imagine a spool of bright green thread”), in isolation and against a blank background. The novel, fictitious scenes (e.g. “imagine standing on the crowded platform of a train station”) were to be as vivid and life-like as possible, and they were instructed to imagine all aspects of the scenes (such as the surrounding environment, how it looked, felt, smelled, and sounded). For the scene task it was repeatedly emphasised that they should not simply recall an experience they have had, but they should create something new, and for objects not to simply bring to mind a familiar object, but again to imagine an entirely new object. Key task instructions were reinforced between each of the scanning sessions.

**Fig. 2:**
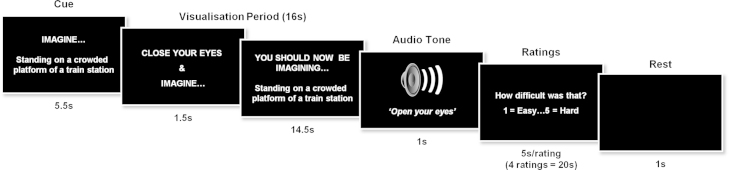
Timeline of an example scene construction trial from Jon's fMRI study. See Section 2 for full details.

We used a very similar paradigm for Jon and P01 (Mullally et al., 2012). Three minor adaptations were made to assist the amnesic patients. First, the scenario cue appeared on the screen throughout the visualisation period, accompanied by the words ‘You should now be imagining [cue]’, in case the patient opened his eyes and could not recall the task (Fig. 2). Second, for the ratings, instead of one-word cues being used (e.g. ‘Difficulty?’) a full question was used (‘How difficult was that?’), with 0.5 s extra added per rating to allow for the additional reading. Third, just two of the experimental tasks were included from the original Hassabis et al. (2007) protocol, namely, constructing scenes and constructing single acontextual objects for the first time in the scanner. The other tasks—recall of recent autobiographical memories, recall of previously viewed objects, recall of previously imagined scenes and recall of previously imagined objects could not be included given the patients’ amnesia.

Jon and P01 therefore performed an experiment with two main experimental conditions (imagining scenes and imagining objects). Each of these conditions had 20 trials. Ten baseline control trials (imagining a white cross on a black background) were also included, yielding a total of 50 trials presented across three scanning sessions. Prior to scanning, Jon (and P01) received extensive training to ensure he was thoroughly familiarized with all aspects of the task, including task cues, instructions, timings and key presses, and to confirm that he could retain the instructions during the scanning session. The spatial coherence rating collected at the end of each experimental trial provided an important internal check, enabling us to verify that Jon had retained the task cue throughout the trial (i.e. if the trial was an object trial, then it should be rated low on the coherence scale and as ‘an isolated object’, whereas if the cue described a scene, then the trial should be rated higher and be considered ‘a contiguous scene’).

Immediately following scanning, participants were thoroughly debriefed. Jon and P01 were asked a number of additional questions to attempt to ascertain how they had performed the task under the constraints imposed by their amnesia. For example, they were asked to describe what they had been doing during scanning, and to report the task instructions. In addition, three of the scene cues and three of the object cues that had been presented during scanning were re-presented, one at a time (two scene/object cues were re-presented to controls). All participants were then asked to imagine the scene (or object) and to provide a description of this out loud. This provided an indication of the participants’ scene construction abilities. In the case of Jon and P01, they were then asked to describe how they went about constructing a scene in their mind's eye, and were asked to reflect on how effortful they found scene construction.

### 2.3 Behavioural data analysis

Data are presented as mean values±SD. Statistical significance was calculated by looking at differences in the ranked position order of Jon's ratings for the scene trials and the object trials (Mann–Whitney *U* test). All tests performed were two-tailed and differences were considered statistically significant at *p*<0.05.

### 2.4 Scanning parameters and preprocessing

T2^⁎^-weighted echo planar images (EPI) with blood oxygen level-dependent (BOLD) contrast were acquired on a 1.5 T Siemens AG (Erlangen, Germany) Sonata MRI scanner. Scanning parameters were selected to achieve whole brain coverage: 45 oblique axial slices angled at 30 degrees in the anterior–posterior axis, 2 mm thickness (1 mm gap), repetition time 4.05 s, slice time 90 ms, TE 50 ms, field of view 192 mm, 64×64 matrix, in-plane resolution 3×3 mm. The first 6 ‘dummy’ volumes from each session were discarded to allow for T1 equilibration effects. Field maps were acquired with a standard manufacturer's double echo gradient echo field map sequence (short TE=10 ms, long TE=14.76 ms; whole brain coverage; voxel size, 3×3×3 mm). A T1-weighted structural scan was also acquired with 1 mm isotropic resolution. Data were analysed using the statistical parametric mapping software SPM8 (www.fil.ion.ucl.ac.uk/spm). The Hassabis et al. (2007) control participants’ data were re-analysed in SPM8 to allow for direct comparison with Jon's data. Spatial preprocessing consisted of realignment and unwarping (using field maps), normalization to a standard EPI template in Montreal Neurological Institute (MNI) space with a resampled voxel size of 3×3×3 mm, and smoothing using a Gaussian kernel with full width at half maximum of 8 mm.

### 2.5 fMRI data analysis

After preprocessing, statistical analysis was performed using the general linear model. The experiment had two main imagining conditions (scenes and objects) and one baseline control (fixation cross) condition. We modelled the time period from the start of the visualisation period (from the ‘close your eyes and imagine’ cue) until the end of the visualisation period as a boxcar function of 16 s duration. This was convolved with the canonical haemodynamic response function to create regressors of interest. Jon's movement parameters were included as regressors of no interest and the subject-specific parameter estimates pertaining to each regressor (betas) were calculated for each voxel. First level contrasts were performed on these parameter estimates. As in Hassabis et al. (2007), we report the fMRI results at a voxel-level threshold of *p*<0.001 whole brain uncorrected (minimum cluster size of 5 voxels). We report all areas activated at this threshold.

A direct comparison of Jon's fMRI data with that of the control participants (*n*=21) was also performed. First, we identified regions that were more active in the control participants than in Jon. We therefore subtracted Jon's ‘scenes>objects’ contrast image from each of the control's corresponding ‘scenes>objects’ contrast images, and entered the resulting differences images into a one sample *t*-test. This enabled us to identify all of the regions that were more active in the control group than in Jon during the scene construction trials (see http://www.mrc-cbu.cam.ac.uk/people/rik.henson/personal/Henson_Singlecase_06.pdf). Next, we reversed the procedure and subtracted each of the control's contrast images from Jon's contrast image (for the ‘scenes>objects’ comparison). Again the resulting twenty one difference images were entered into a one sample *t*-test. Due to the fixed-effects nature of this analysis we report all areas activated at a voxel-level threshold of *p*<0.05 (whole-brain FWE corrected; minimum cluster size of 5 voxels).

We then compared Jon's scene construction network with that of patient P01 (Mullally et al., 2012). We did this by comparing the ‘P01-Controls’ difference images [constructed by individually subtracting each of the control's contrast images (for the ‘scenes>objects’ comparison) from P01’s scenes>objects contrast images] to ‘Jon-Controls’ difference images [constructed by individually subtracting each of the control's contrast images (for the ‘scenes>objects’ comparison) from Jon's scenes>objects contrast images] using a two samples *t*-test. As before, we report all areas activated at a voxel-level threshold of *p*<0.05 (whole-brain FWE corrected; minimum cluster size of 5 voxels).

Finally, using anatomical masks for the hippocampus, parahippocampal cortex and retrosplenial cortex we extracted the average parameter estimates within each of these regions for the scenes>objects contrast for Jon, P01 and each of the 21 control participants. The anatomical masks were delineated by an experienced researcher not involved in the project on an averaged structural MRI brain scan from a different set of *n*=30 control participants and segmentation was guided by Duvernoy (1999) and Vann, Aggleton, and Maguire (2009). The masks were visually inspected on each participant to ensure that no CSF, or grey/white matter from neighbouring regions was included.

## 3 Results

### 3.1 Behavioural data

#### 3.1.1 Ratings

After each visualisation period throughout the scanning session Jon rated the imagined objects and scenes in terms of their difficulty, vividness, coherence and similarity to a memory (using a five point scale). In terms of trial difficulty, Jon did not report finding the scenes more difficult to imagine than the objects (objects: mean=1.3, SD=0.98; scenes: mean=1.55, SD=0.94; *U*=163.5, *Z*=−1.42, *P*=0.16). He did, however, rate his constructed objects as significantly more vivid than his constructed scenes (objects: mean=4.8, SD=0.7; scenes: mean=4.25, SD=0.7; *U*=122.5, *Z*=−2.6, *P*<0.01), perhaps betraying his difficulty imagining truly vivid scenes. He tended to rate the scenes as more like memories than the imagined objects (objects: mean=2.73, SD=1.39; scenes: mean=3.5, SD=1.47; *U*=134.5, *Z*=−1.82, *P*=0.07) although this difference did not reach statistical significance. Most importantly, however, Jon rated the scenes as ‘coherent’ (mean=4.75, SD=1.0) and the objects as significantly less ‘scene-like’ (mean=2.53, SD=1.85; *U*=43, *Z*=−3.72, *P*<0.01). This shows that, despite his amnesia, Jon managed to successfully retain the task instructions throughout the scanning session and was able to recall the cue beyond the 16 s visualisation period. Finally, following the baseline control trials, Jon reported that he was able to maintain a high level of focus (mean=3.88, SD=1.25).

Table 1 shows Jon's behavioural ratings alongside those of the control participants and P01. They were highly comparable in terms of difficulty, vividness and coherence. Although Jon finds constructing scenes effortful and does so bit by bit, he is intelligent and well-practised at it, so it is perhaps not surprising that his ratings were similar to the control subjects. On the other hand, as noted above, despite being amnesic Jon rated his imagined scenes (and objects) as being more similar to memories compared to the control subjects. This highlights a potential caveat. As we have pointed out elsewhere (Cooper et al., 2011), given that patients with developmental amnesia sustain such early hippocampal damage and may never have known what it is like to truly imagine a scene or event, then in the absence of any comparator, their ratings may be more difficult to interpret. For instance, Jon may have rated his constructions as similar to memories because they comprised semantic information, and this may be the only type of information to which he had access. This situation is in contrast to amnesic patients whose hippocampal damage is acquired in adulthood who, in our experience, are acutely aware that some of their abilities have changed post-lesion and make their ratings accordingly.

**Table 1:** Behavioural ratings.

	Difficulty	Vividness	Coherence	Memory
SCENES				
Controls	1.85 (0.45)	3.91 (0.64)	3.91 (0.58)	1.94 (0.33)
Jon	1.55	4.25	4.75	3.50
P01	1	4.95	5	2.9
OBJECTS				
Controls	1.65 (0.44)	4.09 (0.43)	1.67 (0.56)	1.74 (0.54)
Jon	1.3	4.8	2.53	2.73
P01	1.4	5	1	1.4

Means and between subjects SDs for the controls (data from Hassabis et al., 2007), means for Jon (see text for within subject SDs) and P01 (see Mullally et al., 2012 for within subject SDs).

#### 3.1.2 Debriefing

In a previous behavioural study (Maguire et al., 2010) we reported on Jon's scene construction performance in detail and provided examples of his imagined scenes. Overall, he performed comparably to controls on measures of scene content—spatial references, entities present and thoughts/emotions/actions. His score for sensory descriptions was borderline impaired, which may betray the effortful nature of his visualising. His rating of the spatial coherence of constructed scenes was also indistinguishable from controls. In the current fMRI study, we focussed on checking that his scene construction remained at a similar level. After scanning, Jon was presented with three scene and three object cues that had been given during scanning and was asked to imagine each one this time out loud. As before, he was able to describe detailed and coherent scenes. Of note, however, he characterised the scene construction process as effortful, and as one that he “has had to work at”. Jon reported that he does not initially visualise “a definite image that has everything in it”, rather he constructs the scene bit by bit, and the scene “is not an instant picture”. In addition, he stated that he does not visualise scenes in everyday life and that he would not describe himself as a visualising person in relation to scenes. P01’s post-scan testing (detailed in full in Mullally et al., 2012) confirmed the preserved nature of his scene construction. In contrast to Jon, however, P01 noted that “most of the scene comes in one shot” and described the addition of the extra details as akin to “colouring in a colour book.” He noted that he does not find scene construction an effortful process but something that comes quite naturally to him.

### 3.2 Neuroimaging data

#### 3.2.1 Jon's scene construction network

**Fig. 3:**
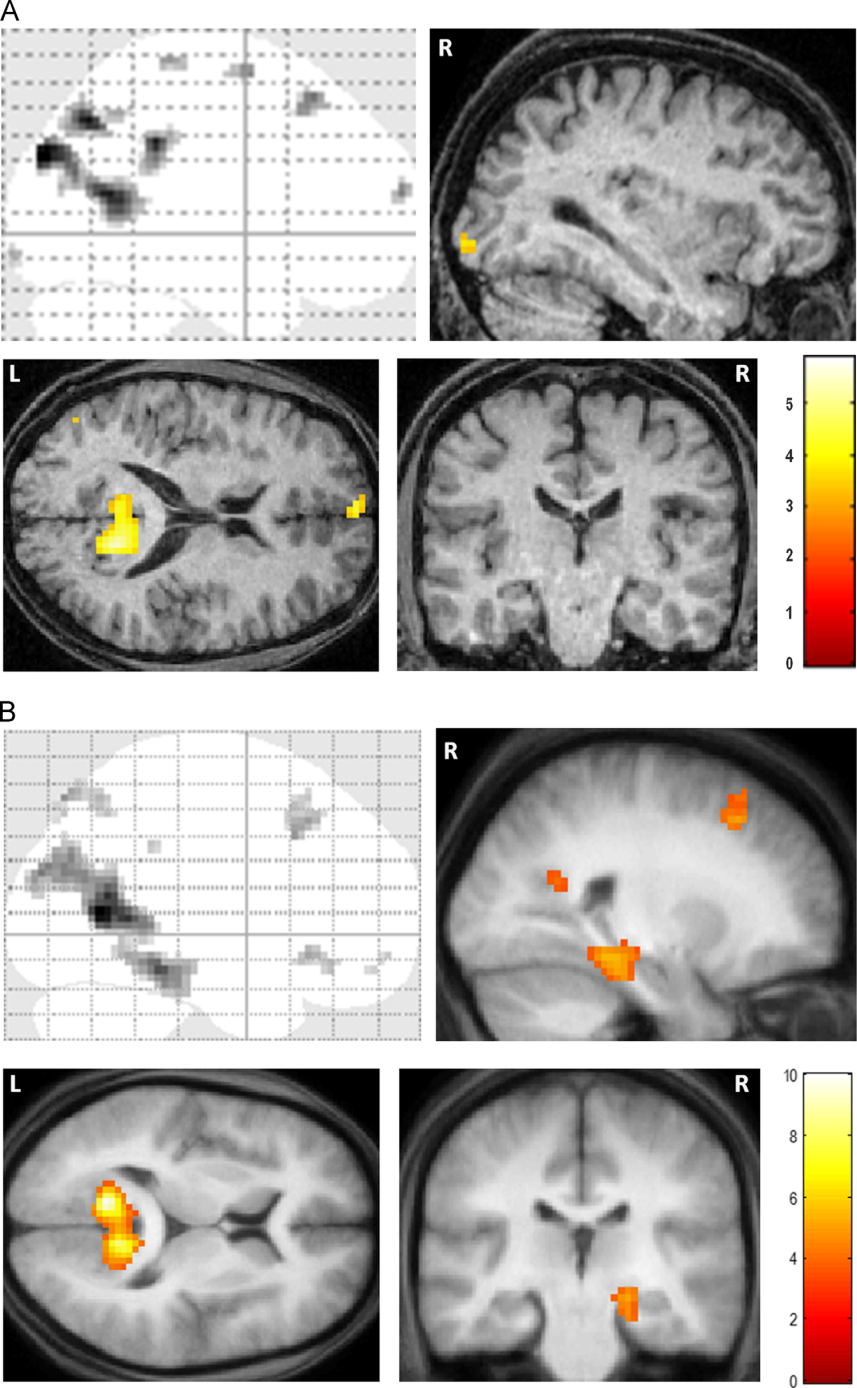
fMRI results. (A) Brain areas more active for constructing scenes compared to imagining single acontextual objects in patient Jon. The upper left panel shows the sagittal image from a “glass brain” which enables one to appreciate activations at all locations and levels in the brain simultaneously. Activations are shown on sagittal (upper right panel), axial (lower left panel) and coronal (lower right panel) images from Jon's structural MRI scan at a threshold of *p<*0.001 (whole brain, uncorrected). The colour bar indicates the *z*-scores associated with each voxel. L=left side of the brain, R=right side of the brain. (B) The same contrast in control participants (data from Hassabis et al., 2007) shown on the averaged structural MRI scan of those participants at a threshold of *p*<0.001 (whole brain, uncorrected).

**Fig. 4:**
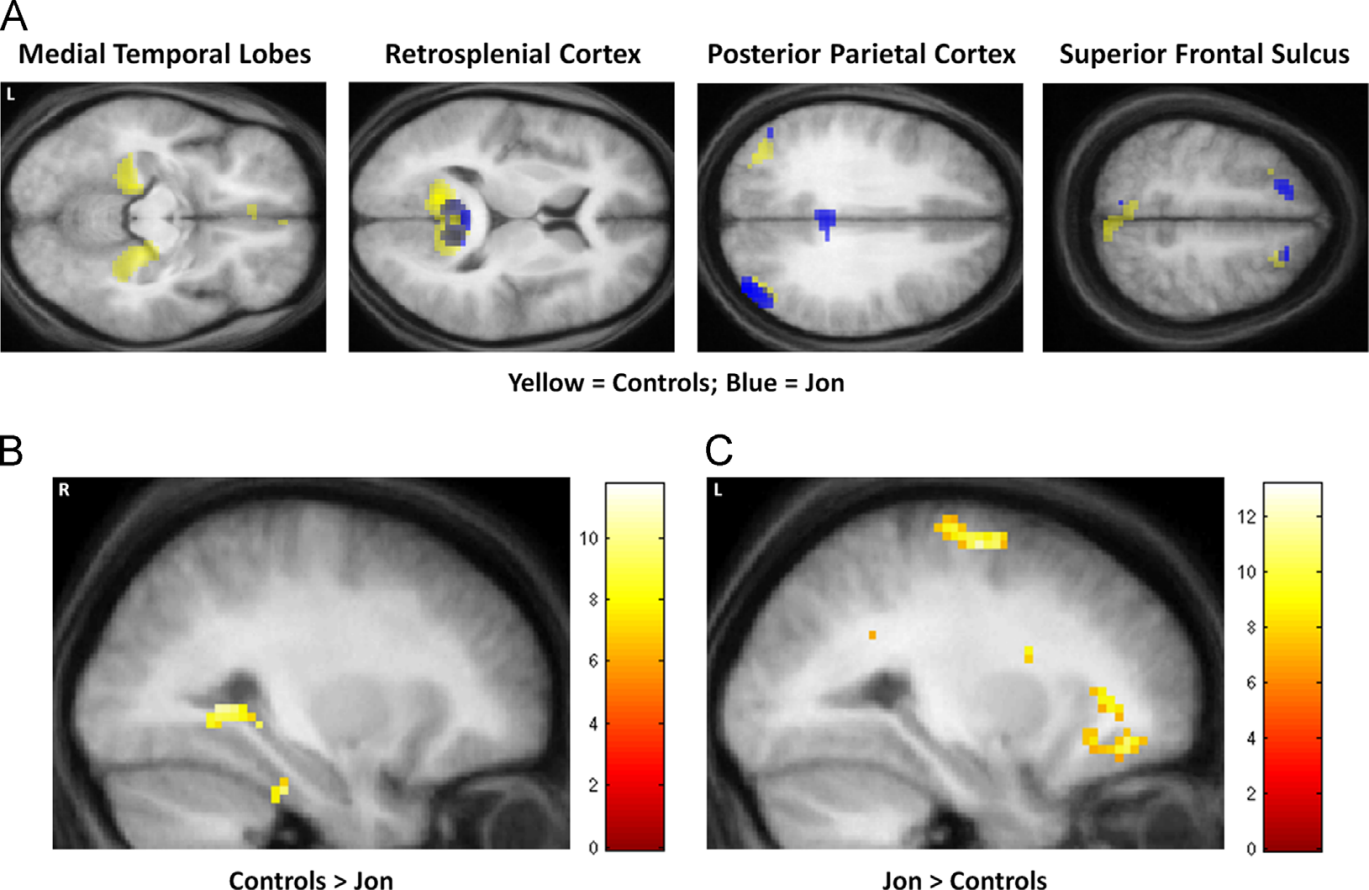
Comparisons between Jon and the control participants. (A) The overlap between Jon and control participants—blue blobs are activations from Jon and yellow blobs are the activations for the control participants from Hassabis et al. (2007). Data are shown at *p*<0.005 for display purposes on axial images from the averaged structural MRI scan of the control participants. (B) The regions activated more by controls than by Jon during scene construction at a threshold of *p<*0.05 (FWE-corrected, whole brain) shown on a sagittal slice from the averaged structural MRI scan of the control participants. (C) The regions activated more by Jon than the controls during scene construction at a threshold of *p<*0.05 (FWE-corrected, whole brain) shown on a sagittal slice from the averaged structural MRI scan of the control participants. The colour bars indicate the *z*-scores associated with each voxel. L=left side of the brain, R=right side of the brain. (For interpretation of the references to color in this figure legend, the reader is referred to the web version of this article.)

In order to appreciate the brain areas engaged when Jon constructed novel fictitious scenes, we compared activity associated with the imagination of these scenes relative to the imagination of single acontextual objects (scenes>objects). We observed increased activity in VMPFC, bilaterally in the superior frontal sulci, posterior cingulate, retrosplenial and posterior parietal cortices (Table 2 and Fig. 3A). Thus, Jon appeared to engage many of the regions typically activated when control participants (such as those reported in Hassabis et al., 2007) construct novel scenes relative to novel objects (Table 2 and Fig. 3B). The striking overlap between Jon's activation pattern (in retrosplenial, posterior parietal and frontal regions) and that associated with the Hassabis et al. (2007) controls is highlighted in Fig. 4A, where Jon's activation clusters clearly lie within the controls’ scene construction network.

**Table 2:** FMRI results.

Region	Peak coordinate (*x*, *y*, *z*)	*Z*
Jon		
*Scenes>objects*		
Left ventromedial prefrontal cortex	−3, 65, 16	4.09
Left superior frontal sulcus	−18, 29, 55	4.19
Right superior frontal sulcus	21, 29, 55	4.12
Left middle frontal gyrus	−21, −1, 67	3.97
Left precentral gyrus	−18, −31, 70	3.84
Right posterior cingulate cortex	6, −37, 37	5.07
Left posterior cingulate cortex	−3, −31, 40	3.67
Right retrosplenial cortex	15, −55, 16	5.15
Left retrosplenial cortex	−3, −55, 16	4.15
Right precuneus	−3, −64, 46	5.26
Left precuneus	15, −67, 46	4.82
	18, −52, 49	3.70
Right posterior parietal cortex/angular gyrus	42, −82, 31	5.58
Left posterior parietal cortex/angular gyrus	−51, −70, 28	3.76
	−48, −76, 19	3.59
Right superior occipital cortex	21, −103, 4	3.70
Right inferior occipital cortex	33, −97, −8	3.63
Control participants^a^		
*Scenes>objects*		
Right ventromedial prefrontal cortex	3, 24, −9	4.27
Right superior frontal sulcus	27, 27, 45	4.42
Right middle temporal cortex	57, −6, −24	3.70
Right hippocampus	21, −24, −12	3.86
Left parahippocampal gyrus	−18, −36, −15	4.28
Right parahippocampal gyrus	33, −42, −12	4.43
Left retrosplenial cortex	−12, −60, 9	6.08
Right retrosplenial cortex	12, −57, 15	5.52
Right precuneus	9, −57, 48	3.91
Left posterior parietal cortex	−48, −78, 24	4.75
Right posterior parietal cortex	45, −66, 24	4.75

a Data from Hassabis et al. (2007). Note that the scenes>objects analysis reported here for the control participants (unlike the contrast reported in Hassabis et al., 2007) is restricted to items newly-imaged in the scanner, in order to be identical to the tasks performed by Jon.

In addition to these overlapping regions of activity, what is also striking is that Jon did not appear to activate any MTL structures while constructing his scenes (Table 2, Fig. 3A). In order to explore this difference further, we first lowered the threshold for statistical significance to 0.005 (whole-brain uncorrected) for Jon's scenes>objects contrast to check for any sub-threshold voxels of activity within these regions. Two small clusters of activity were observed in right (30, −34, −17; *Z*=2.98) and left (−21, −22. −22; *Z*=2.99) parahippocampal cortex. However, no increase in activity was observed in either hippocampus. These results therefore differ from those observed in control participants, who show extensive parahippocampal and right hippocampal activity (Fig. 3B).

#### 3.2.2 Comparisons between Jon and the control participants

To assess this apparent difference between Jon's scene construction network and that of controls, we directly compared Jon's data (scenes>objects) with that of the control participants (Table 3). As anticipated, the control participants activated their right hippocampus more than Jon (Fig. 4B), as well as right perirhinal cortex, and VMPFC. Jon, on the other hand, activated his right retrosplenial cortex, bilateral posterior cingulate cortex, left lateral orbital gyrus, left precentral gyrus and left middle frontal gyrus (Fig. 4C) more than controls. This suggests that Jon, unlike controls, was constructing his scenes in a non-hippocampal fashion, perhaps relying upon components of the scene construction network (such as the retrosplenial cortex), and the recruitment of additional frontal regions, to a greater extent than control participants.

**Table 3:** Comparison of Jon's fMRI data (scenes>objects) with controls and P01.

Region	Peak Coordinate (*x*, *y*, *z*)	*Z*
*Controls>Jon*		
Ventromedial prefrontal cortex	0, 20, −11	5.39
Right hippocampus	30, −37, −5	5.60
Right perirhinal cortex	30, −28, −29	5.72
*Jon>controls*		
Left middle frontal gyrus	−21, −1, 67	5.96
Left lateral orbital gyrus	−27, 41, −11	6.00
Left precentral gyrus	−18, −34, 70	6.56
Right retrosplenial cortex	12, −46, 16	6.17
Right posterior parietal cortex	9, −37, 34	6.18
Left posterior parietal cortex	−3, −37, 28	5.41
*P*01*>Jon*		
Right hippocampus	36, −28, −11	7.84
	30, −46, −2	6.98
	30, −37, −5	6.70
*Jon>P*01		
Right dorsolateral prefrontal cortex	60, 14, 25	7.77
Left dorsolateral prefrontal cortex	−57, 5, 40	7.56
Right superior frontal gyrus	18, 35, 37	>8.0
Left precuneus	−3, −61, 46	7.48
Left inferior temporal gyrus	−60, −10, −26	7.59
Right visual cortex	6, −103, 1	7.72
Right lateral occipital cortex	48, −49, −14	>8.0

**Fig. 5:**
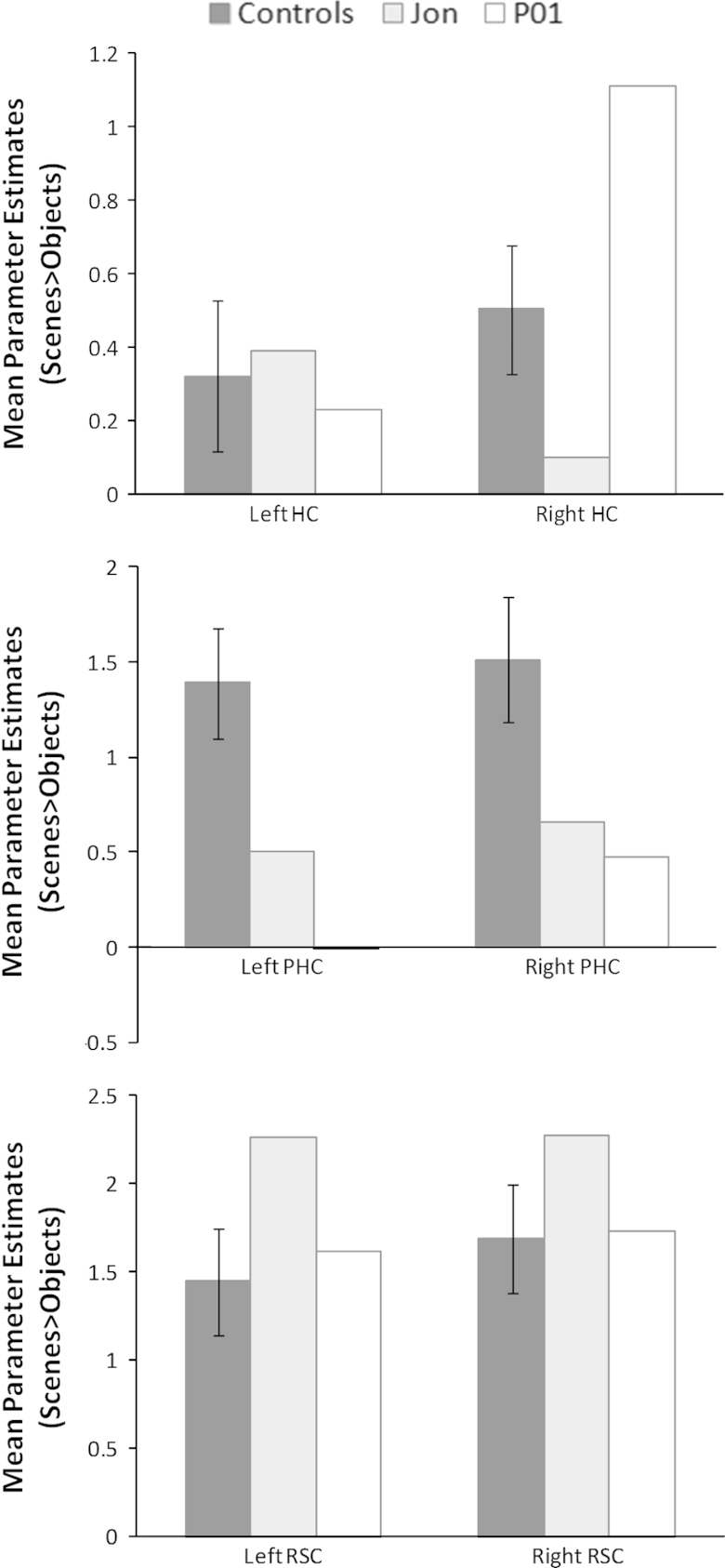
The average parameter estimates from three regions (defined using anatomical masks – see Section 2) within the core network for Jon, P01 and the control participants: the hippocampus (HC), parahippocampal cortex (PHC) and retrosplenial cortex (RSC), for the scenes – objects contrast.

Fig. 5 shows the average parameter estimates from three regions (defined using anatomical masks – see Section 2) within the core network for Jon and the control participants – the hippocampus, parahippocampal cortex and retrosplenial cortex for the scenes – objects contrast. This confirms that the left hippocampus, even in controls participants was not significantly engaged by the task compared to the right hippocampus (as reported by Hassabis et al., 2007), and that Jon's right hippocampus was much less engaged than the right hippocampus of controls. By contrast, Jon's retrosplenial cortex was more active compared with the control subjects.

**Fig. 6:**
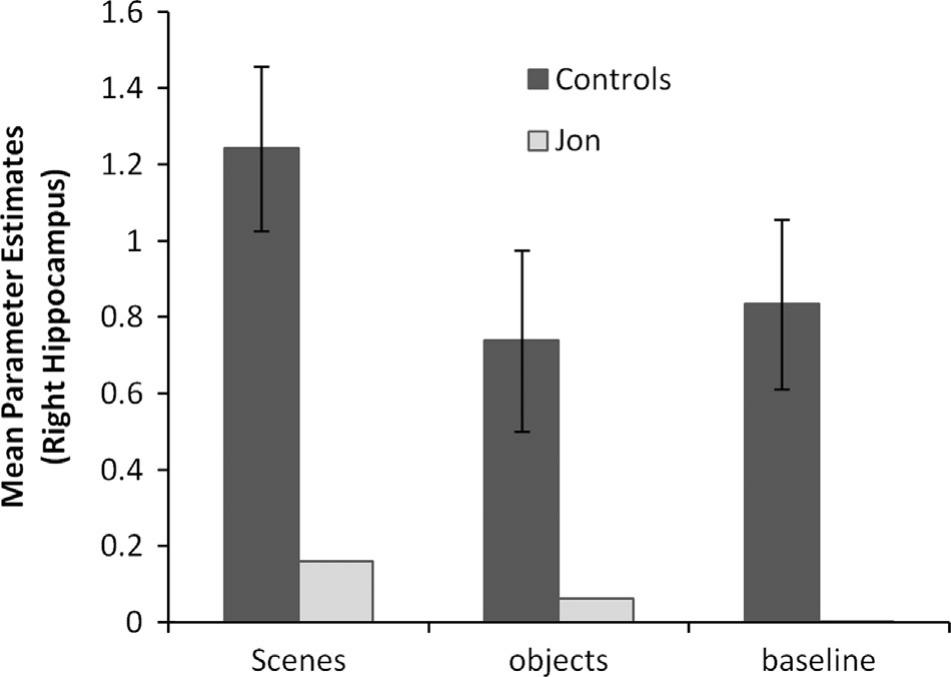
The average parameter estimates (scenes-objects contrast) within the right hippocampus (delineated with an anatomical mask—see Section 2) for scene, object and fixation baseline conditions.

We also examined the average parameter estimates within the right hippocampus (delineated with an anatomical mask – see Section 2) to determine the pattern of activity for scene, object and fixation baseline conditions (Fig. 6). It is clear that there was very little activity in Jon's right hippocampus for any condition, and significantly much less than that of the control participants.

#### 3.2.3 Comparisons between Jon and P01

**Fig. 7:**
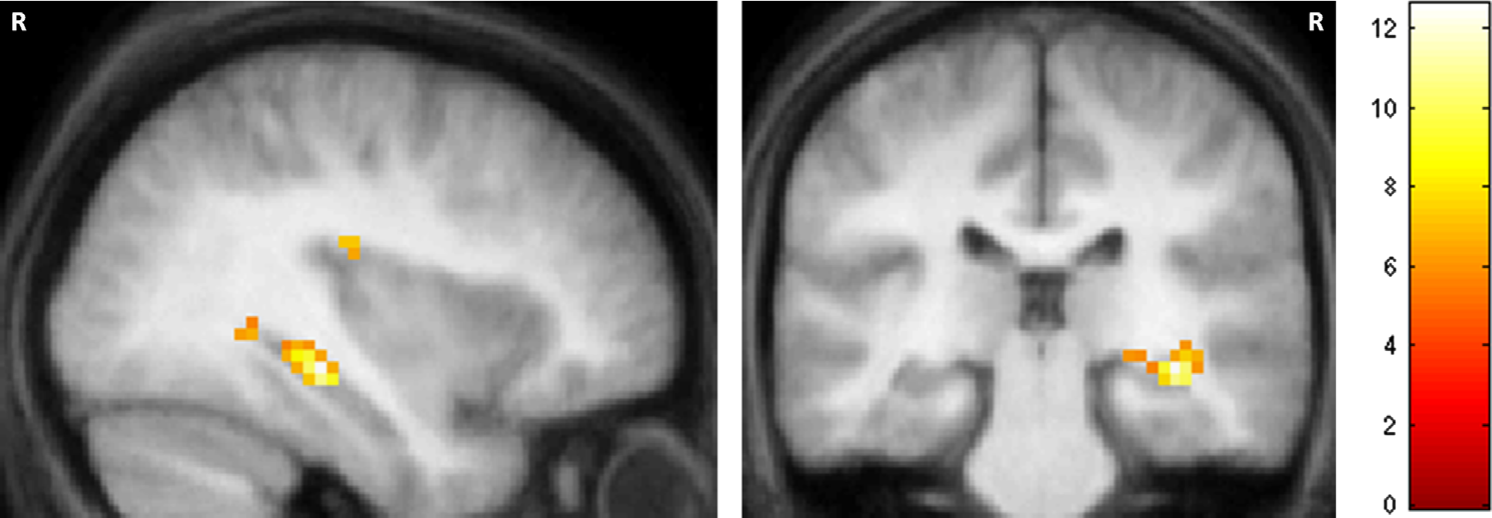
Comparison between Jon and P01. P01 activated his right hippocampus significantly more than Jon. Results are displayed at a threshold of *p<*0.05 (FWE-corrected, whole brain) on a sagittal (left) and coronal slice (right) from the averaged structural MRI scan of the control participants. The colour bar indicates the *z*-scores associated with each voxel. R=right side of the brain.

Patient P01 also activated his right hippocampus while constructing scenes (reported in detail in Mullally et al., 2012). In summary, we found that scene construction in P01 was associated with increased activity in a set of brain areas including medial temporal, retrosplenial and posterior parietal cortices, that overlapped considerably with the regions engaged in the control participants performing the same task. Most notably, the remnant of P01’s right hippocampus exhibited increased activity during scene construction compared with the control participants. We therefore hypothesised that P01 would show significantly greater hippocampal activation than Jon. As illustrated in Fig. 7 and Table 3, this is what we found (see also Fig. 5), with P01’s right hippocampus significantly more active than Jon's as he constructed novel scenes. Jon activated several regions more than P01 (Table 3); primary peaks: right superior frontal gyrus, right lateral occipital cortex; right dorsolateral prefrontal cortex, left dorsolateral prefrontal cortex, right visual cortex, left inferior temporal gyrus and left precuneus.

## 4 Discussion

Patients with DA resulting from early bilateral hippocampal damage such as Jon have consistently been found to retain the ability to mentally construct scenes (Cooper et al., 2011, Hurley et al., 2011, Maguire et al., 2010). This stands in contrast to patients with amnesia whose hippocampal damage was sustained in adulthood who typically show a scene construction impairment (e.g. Hassabis et al., 2007, Mullally et al., 2012). Until now, it has been uncertain whether the form of scene construction expressed by patients with DA depends upon the hippocampus. Using fMRI we investigated the neural network supporting the well-characterised patient Jon's scene construction, and whether this would include activation of his remnant hippocampal tissue. When Jon engaged in scene construction he activated many of the regions known to be associated with imagining scenes and imagining the future in control participants (e.g. Hassabis et al., 2007, Addis et al., 2007, Summerfield et al., 2009, Summerfield et al., 2010), including VMPFC, posterior cingulate, retrosplenial and posterior parietal cortices. Critically, however, activity was not increased in the key region of interest, Jon's remnant hippocampal tissue, even when more liberal statistical thresholds were applied. Direct comparisons with a group of control participants and also another single case, patient P01 (Mullally et al., 2012), confirmed that they activated their right hippocampus more than Jon. By contrast, Jon engaged his retrosplenial cortex, posterior parietal cortex, and several additional areas within frontal cortex more than controls.

A failure to observe increased hippocampal activity associated with Jon's scene construction does not mean that there was no activity in Jon's hippocampus as he performed this task—we acknowledge that absence of evidence is not evidence of absence. However, our findings show that activity in this region was insufficiently strong to be detected using the same tasks, imaging techniques and statistical analyses that were able to detect increased hippocampal engagement in controls and also in another amnesic patient with significant hippocampal volume loss, P01. Similarly, when directly compared to the controls and P01, Jon's hippocampal activity was significantly less.

This difference between the control subjects and Jon in levels of hippocampal activity only emerged in the posterior hippocampus, which does not appear to overlap with the more mid-hippocampal region activated strongly in controls during the scene construction task. It could be that although Jon did not engage this mid-hippocampal region above the statistical threshold, he perhaps activated it to a level that is not different from controls. However, the parameter estimate plots from across the hippocampus show that Jon's right hippocampus was essentially non-responsive. Moreover, as we have noted before in relation to P01 (Mullally et al., 2012), any intra-hippocampal considerations in patients with significant hippocampal damage must be treated with caution, because while it is possible to assign coordinates in stereotactic space to fMRI activations, what exactly this means for a grossly atrophied hippocampus is uncertain. Jon's atrophy is along the entire length of the hippocampus. Attempting to infer localisation of function within the hippocampus in this context may be futile, as anterior/posterior distinctions might not now be observed. We conclude, therefore, that while Jon's scene construction showed a number of commonalities with that of controls and P01, it did not seem to depend on the hippocampus in the same way as scene construction in controls and P01. So how did Jon manage to produce descriptions of scenes without, or with substantially less involvement of, his hippocampus?

Klein, 2012, Klein, 2013, Klein and Lax (2010), Klein et al. (2002) have emphasised that mental simulation is possible using semantic memory. We suggest that the ability of patients with DA to acquire semantic knowledge may be critical in their execution of a form of scene construction. They may use this preserved semantic and world knowledge, a sense of familiarity, and intact reasoning ability about how imagined events logically unfold, to describe scenes, but they do not vividly experience these scenes. Interestingly, Jon reported that he finds scene construction effortful and commented that his scenes are not instant pictures, and that he does not imagine scenes in everyday life. By contrast controls and P01 describe their construction of scenes as automatic. It may therefore be important to draw a distinction between scenes that are effortfully constructed and described, and those that are automatically constructed, truly visualised in the imagination and vividly experienced, with the latter depending on the hippocampus.

Compared to controls, Jon exhibited up-regulation of several areas within the scene construction network, including retrosplenial and posterior parietal cortices, as well as recruiting additional parts of prefrontal cortex. We offer some speculations about the reasons for these increased areas of activity compared with the control participants. The retrosplenial cortex is consistently engaged by a range of tasks that examine episodic memory, imagining the future, spatial navigation, and scene processing (Vann et al., 2009). It has been suggested that its core function may be related to the geometric layout of scenes (Epstein, 2008), or transitioning between egocentric and allocentric perspectives (Vargha-Khadem et al., 1997, Ranganath and Ritchey, 2012). Along with the posterior parietal cortex, this could mean that in his attempt to construct coherent scenes, Jon is over-reliant on spatial processing in these areas as a means of compensating for the lack of spatial input from his hippocampus. Related to this, the retrosplenial cortex has recently been implicated in processing the most stable landmarks in the environment (Auger, Mullally, & Maguire, 2012). Its up-regulation could also index the retrieval of stable items, but which Jon's dysfunctional hippocampus cannot use to construct a spatially coherent scene.

There could be other reasons for the up-regulation/recruitment of medial and lateral parietal and prefrontal cortices regions in Jon. Several of these areas have been linked with semantic processing in a meta-analysis of 120 neuroimaging studies by Binder, Desai, Graves, and Conant (2009). Given that Jon has preserved semantic knowledge, he may be over-reliant upon this when attempting to construct imagined scenes. The prefrontal activations could be related to Jon's effortful attempts and/or strategic reasoning to support his performance on scene construction tasks, compensating to some degree for the absence of hippocampal support. A similar finding was apparent when autobiographical memory was assessed longitudinally during fMRI in a patient with semantic dementia. The patient's recollection was initially supported by the classic autobiographical memory network (Svoboda, McKinnon, & Levine, 2006), including atrophied tissue in hippocampus and temporal neocortex. This was subsequently augmented by up-regulation in ventromedial and ventrolateral prefrontal cortex, right lateral temporal cortex, and medial parietal cortex (Maguire, Kumaran, Hassabis, & Kopelman, 2010).

The data from Jon and the other patients with DA show that one can get reasonably far in describing scene-like entities in the absence of significant hippocampal input. It is perhaps surprising then that patients with amnesia whose hippocampal damage occurred in adulthood do not engage in a similar strategy; after all, they would have accumulated a great deal of semantic and world knowledge prior to sustaining their lesions. Patients with DA, in some cases with perinatal hippocampal damage predating the formation of any memory, may never have had the experience of truly visualising scenes in their imagination. Perhaps most of what they glean is ‘second-hand’, so to speak, from other people's descriptions of scenes. Their default is a semantic system. By contrast, in our experience, patients with adult-acquired hippocampal damage and amnesia are acutely aware of what they have lost. They appreciate that their current attempts to visualise scenes in their imagination is a far cry from their pre-morbid ability. Their default is still an episodic system. Therefore, when asked to construct a vivid scene that can be visualised in the imagination they know, perhaps unlike patients with DA, that they simply cannot comply.

In summary, we have shown that DA patient Jon with perinatally-acquired bilateral hippocampal damage and consequent DA is able to construct and describe scenes but this is not associated with increased hippocampal activity of the magnitude observed in control participants and patient P01. Other patients with DA should be scanned using fMRI to test the reliability of this finding further. In addition, it would be useful to explore the basis on which DA patients make their subjective ratings of their constructed scenes. Our data show that a type of non-hippocampal dependent scene construction is possible, perhaps mediated by semantic memory. However, we argue that the inadequacy of this strategy is revealed when scene construction is required as the basis for functions such as episodic memory and spatial navigation, where the inability to construct and truly visualise scenes in the mind's eye results in significant impairments of the type observed in amnesia.
